# Cellular uptake of proMMP-2:TIMP-2 complexes by the endocytic receptor megalin/LRP-2

**DOI:** 10.1038/s41598-017-04648-y

**Published:** 2017-06-28

**Authors:** Manuel Johanns, Pascale Lemoine, Virginie Janssens, Giuseppina Grieco, Soren K. Moestrup, Rikke Nielsen, Erik I. Christensen, Pierre J. Courtoy, Hervé Emonard, Etienne Marbaix, Patrick Henriet

**Affiliations:** 1 de Duve Institute, Université catholique de Louvain, 1200 Brussels, Belgium; 2 Department of Biomedicine, Aarhus University, 8000 Aarhus, Denmark; 3 CNRS UMR 7369, Matrice Extracellulaire et Dynamique Cellulaire, Université de Reims Champagne-Ardenne, 51687 Reims, France

## Abstract

Matrix metalloproteinases (MMPs) are regulated at multiple transcriptional and post-transcriptional levels, among which receptor-mediated endocytic clearance. We previously showed that low-density lipoprotein receptor-related protein-1 (LRP-1) mediates the clearance of a complex between the zymogen form of MMP-2 (proMMP-2) and tissue inhibitor of metalloproteinases, TIMP-2, in HT1080 human fibrosarcoma cells. Here we show that, in BN16 rat yolk sac cells, proMMP-2:TIMP-2 complex is endocytosed through a distinct LRP member, megalin/LRP-2. Addition of receptor-associated protein (RAP), a natural LRP antagonist, caused accumulation of endogenous proMMP-2 and TIMP-2 in conditioned media. Incubation with RAP also inhibited membrane binding and cellular uptake of exogenous iodinated proMMP-2:TIMP-2. Moreover, antibodies against megalin/LRP-2, but not against LRP-1, inhibited binding of proMMP-2:TIMP-2 to BN16 cell surface. BIAcore analysis confirmed direct interaction between the complex and megalin/LRP-2. Conditional renal invalidation of megalin/LRP-2 in mice resulted in accumulation of proMMP-2 and TIMP-2 in their urine, highlighting the physiological relevance of the binding. We conclude that megalin/LRP-2 can efficiently mediate cell-surface binding and endocytosis of proMMP-2:TIMP-2 complex. Therefore megalin/LRP-2 can be considered as a new actor in regulation of MMP-2 activity, an enzyme crucially involved in many pathological processes.

## Introduction

Matrix metalloproteinases (MMPs) compose a family of Zn^2+^-dependent endoproteases that display a large variety of substrates including extracellular matrix macromolecules, cell-surface receptors, growth factors, cytokines and chemokines^1^. These proteinases play important roles in physiological processes, such as wound repair^2^ and cyclic endometrial remodeling^3^, as well as in the development of various pathologies including cancer^4^.

Matrix metalloproteinases are regulated at both transcriptional and post-transcriptional levels. Mechanisms of activity regulation common to all members of the MMP family include activating cleavage of the latent proenzyme form and inhibition of the active enzyme by tissue inhibitors of metalloproteinases (TIMPs)^5^. In addition, extracellular levels of certain MMPs are regulated by selective internalization and intracellular degradation. In this regard, low-density lipoprotein receptor-related proteins (LRPs) compose a family of endocytic and signaling receptors that exert their activities on a large variety of molecules including proteolytic enzymes^6^. Particularly, LRP-1 regulates the extracellular levels of MMPs and serine-proteinases^7, 8^.

Megalin/LRP-2 shares numerous common ligands with LRP-1, notably the complexes between the plasminogen activator (PA) inhibitor type-1 (PAI-1) with either tissue-type PA (tPA)^9^, pro-urokinase-type PA (pro-uPA) or mature uPA^10, 11^. Moreover, a member of the MMP family, MMP-9 also binds to both LRP-1 and -2^12^. In addition, we previously reported that LRP-1 mediates endocytic clearance of the proMMP-2:TIMP-2 complex^13^, which represents the major form of MMP-2 in biological tissues^14^.

In the present study, we investigated the ability of megalin/LRP-2 to control extracellular levels of MMP-2 and TIMP-2 by endocytosis of the proMMP-2:TIMP-2 complex. For this purpose, we used Brown Norway rat yolk sac carcinoma cells (BN16) that express megalin/LRP-2 but not LRP-1^15, 16^. We also evaluated the physiological relevance of proMMP-2 and TIMP-2 uptake in transgenic mice undergoing renal invalidation of megalin/LRP-2^17^.

## Results

### The LRP competitor, RAP, causes accumulation of proMMP-2 and TIMP-2 in medium conditioned by BN16 cells

**Figure 1:**
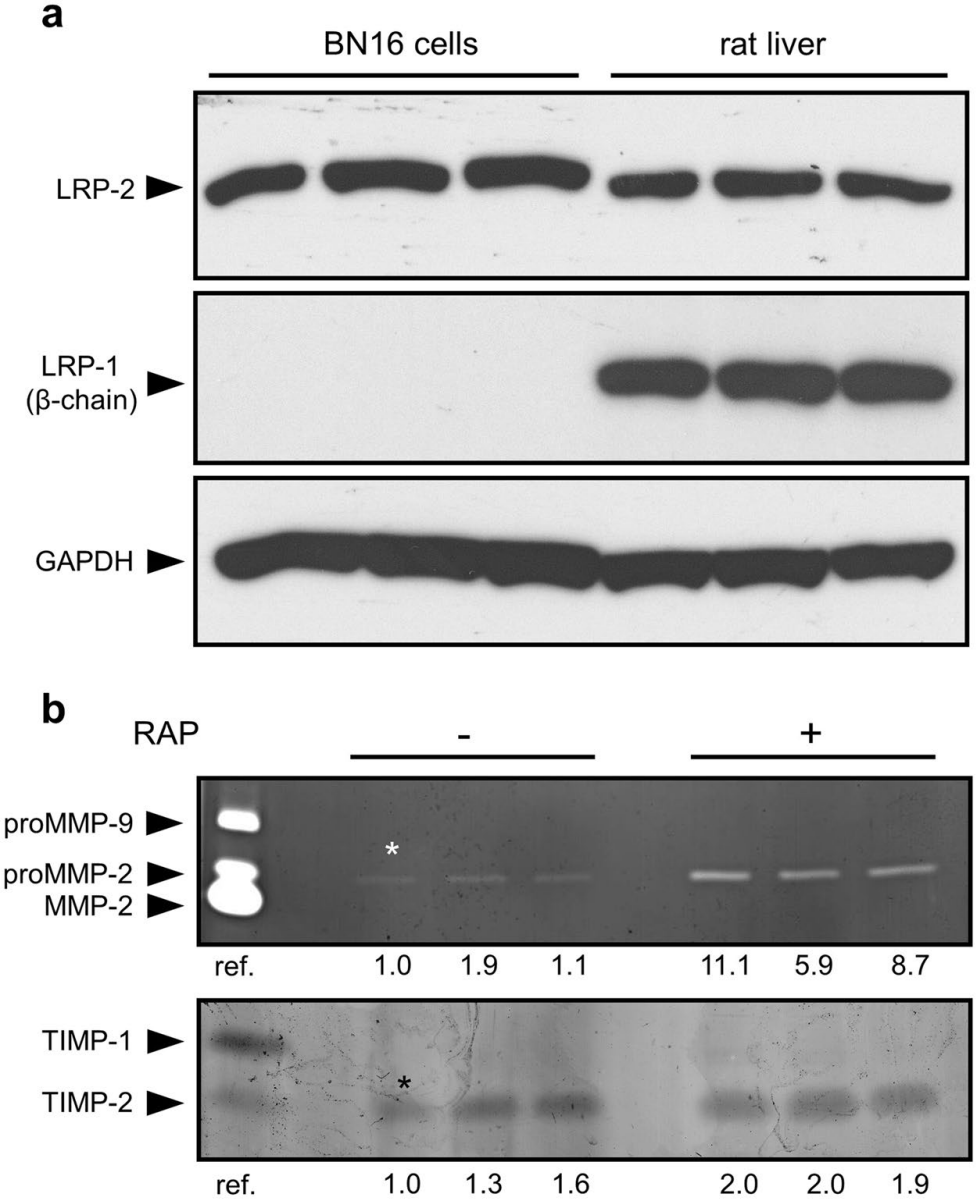
Expression of functional megalin/LRP-2 but not LRP-1 in BN16 cells. (**a**) Expression of megalin/LRP-2 and LRP-1 was evaluated by western blot analysis in BN16 cell extracts (50 μg protein). As positive control, receptor expression was determined in tissue extracts (50 μg protein) from rat liver^40^. Images were cropped for presentation. Additional data without cropping are presented in Supplementary Fig. 1. (**b**) Analysis of media conditioned by BN16 cells in the presence of the LRP competitor, RAP. BN16 cells were cultured for 24 h in serum-free DMEM in the absence or presence of 1 μM RAP. Conditioned media were then collected, and total cell protein was measured using bicinchoninic acid microassay. Top panel, gelatin zymogram of medium conditioned by the equivalent of 5 μg of cell protein. Bottom panel, reverse gelatin zymogram of medium conditioned by the equivalent of 10 μg of cell protein. Representative results of three independent experiments. Reference (ref.) corresponds to medium conditioned by mouse calvarium, as previously reported^41^. Values under the gels indicate the fold-increase by comparison with the first non-treated sample (*). Band area and intensity were measured by using IMAGEJ image analysis software.

We first examined whether proMMP-2 and/or its complex with TIMP-2 could represent a new ligand for megalin/LRP-2. For this purpose, we used rat yolk sac BN16 cells that expressed megalin/LRP-2 (Fig. 1a). As previously reported^16^, we did not detect LRP-1 expression by these cells. In addition, endogenous expression of both proMMP-2 and TIMP-2 could be detected by zymography (Fig. 1b). Incubation of BN16 cells with RAP caused accumulation of both proMMP-2 and, to a lesser extent, TIMP-2 in conditioned media (Fig. 1b). This indicates that a member of the LDL receptor family, all of which are sensitive to ligand binding competition by RAP^18^, mediates the uptake of proMMP-2 and TIMP-2. Since LRP-1 is absent from BN16 cells (Fig. 1a), megalin/LRP-2 appeared to be a good candidate for mediating this process.

### ProMMP-2:TIMP-2 complex binds avidly to megalin/LRP-2

**Figure 2:**
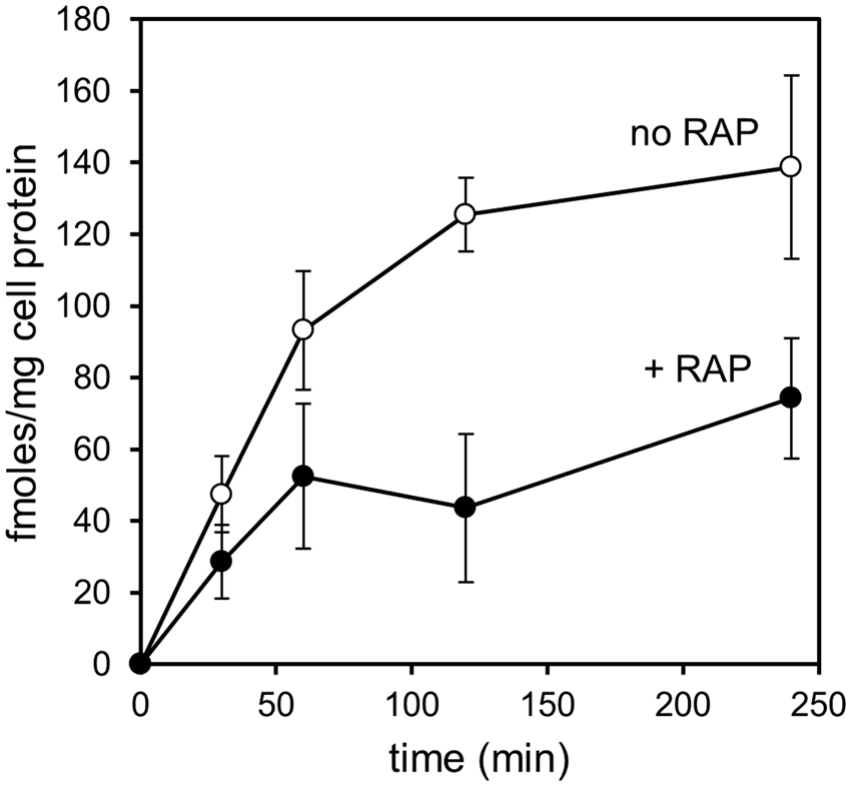
Time-course of cell-surface binding of ^125^I-proMMP-2:TIMP-2 complex. BN16 cells were incubated with 10 nM ^125^I-proMMP-2:TIMP-2 complex at 4 °C for the indicated intervals to allow surface binding, in the absence (open circles) or presence of 1 μM RAP (closed circles), then washed and treated with pronase^®^ for detachment. Surface-bound ^125^I-proMMP-2:TIMP-2 complex was defined as pronase^®^-sensitive radioactivity. Values are means ± S.D. of three dishes. This experiment was performed twice with similar results.

**Figure 3:**
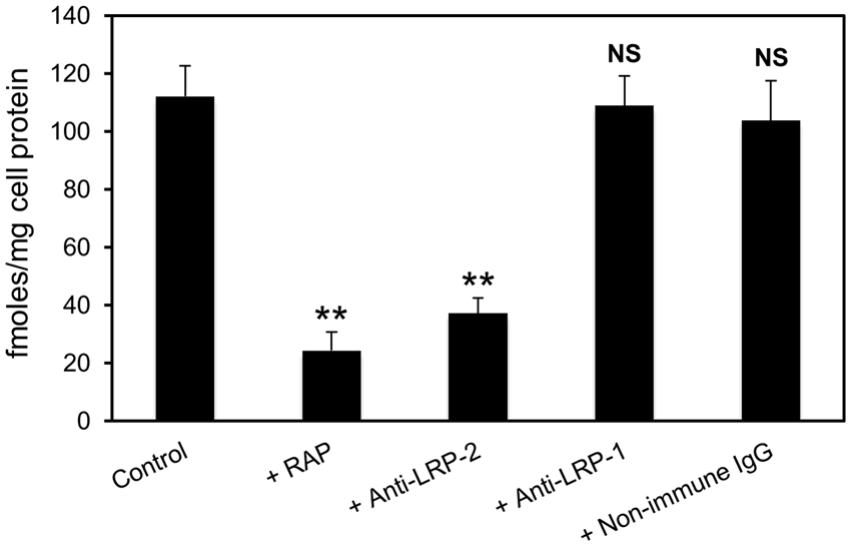
Blocking of megalin/LRP-2 but not LRP-1 inhibits membrane binding of proMMP-2:TIMP-2 complex. BN16 cells were incubated with 10 nM ^125^I-proMMP-2:TIMP-2 complex at 4 °C for 2 h to allow surface binding, in the absence or presence of 1 μM RAP, anti-megalin/LRP-2 IgG (100 μg/ml), anti-LRP-1 IgG (100 μg/ml) or non-immune IgG (100 μg/ml). After 2 h, surface-bound tracer was measured as in Fig. 2. Values are means ± S.D. of three dishes. **p < 0.001; NS, not significant, *vs* control, using Student’s t test.

As MMP-2 is present in biological tissues and fluids mostly in its latent pro-form complexed with its specific inhibitor, TIMP-2^14^, we generated a ^125^I-radiolabelled proMMP-2:TIMP-2 complex for assessing the role of megalin/LRP-2 in the clearance of the complex by BN16 cells. A time-course study indicated that cell-surface binding of ^125^I-proMMP-2:TIMP-2 approached equilibrium after 120 min of incubation at 4 °C and was competitively inhibited by RAP (Fig. 2). As expected, binding was equally blocked by anti-megalin/LRP-2 antibodies but not by non-immune IgG (Fig. 3), suggesting a predominant role of megalin/LRP-2 in this process. As a specificity control, blocking anti-LRP-1 antibodies did not impair binding of ^125^I-proMMP-2:TIMP-2 to BN16 cells.

To assess whether proMMP-2:TIMP-2 complex can directly interact with megalin/LRP-2, we performed surface plasmon resonance analysis using megalin/LRP-2 immobilized on a BIAcore sensor chip. Data in Table 1 revealed a direct and strong interaction between proMMP-2:TIMP-2 complex and megalin/LRP-2, with a *K*_*D*_ value in the 10^−8^ M range.

**Table 1:** Surface plasmon resonance data for direct binding of proMMP-2:TIMP-2 complex to immobilized megalin/LRP-2.

	Complex concentrations (nM)	*k*_*a*_ (0.10^4^ M^−1^ s^−1^)	*k*_*d*_ (0.10^−4^ s^−1^)	*K*_*D*_ (nM)
Experiment 1	25 – 50 – 100 - 250	2.26	4.19	18.6
Experiment 2	10 – 20 – 50 - 100	1.60	2.27	14.2

The equilibrium constants of dissociation (*K*_*D*_) were calculated from the association (*k*_*a*_) and dissociation (*k*_*d*_) rate constants.

### Megalin/LRP-2 mediates clearance of ^125^I-proMMP-2:TIMP-2 complex in BN16 cells

**Figure 4:**
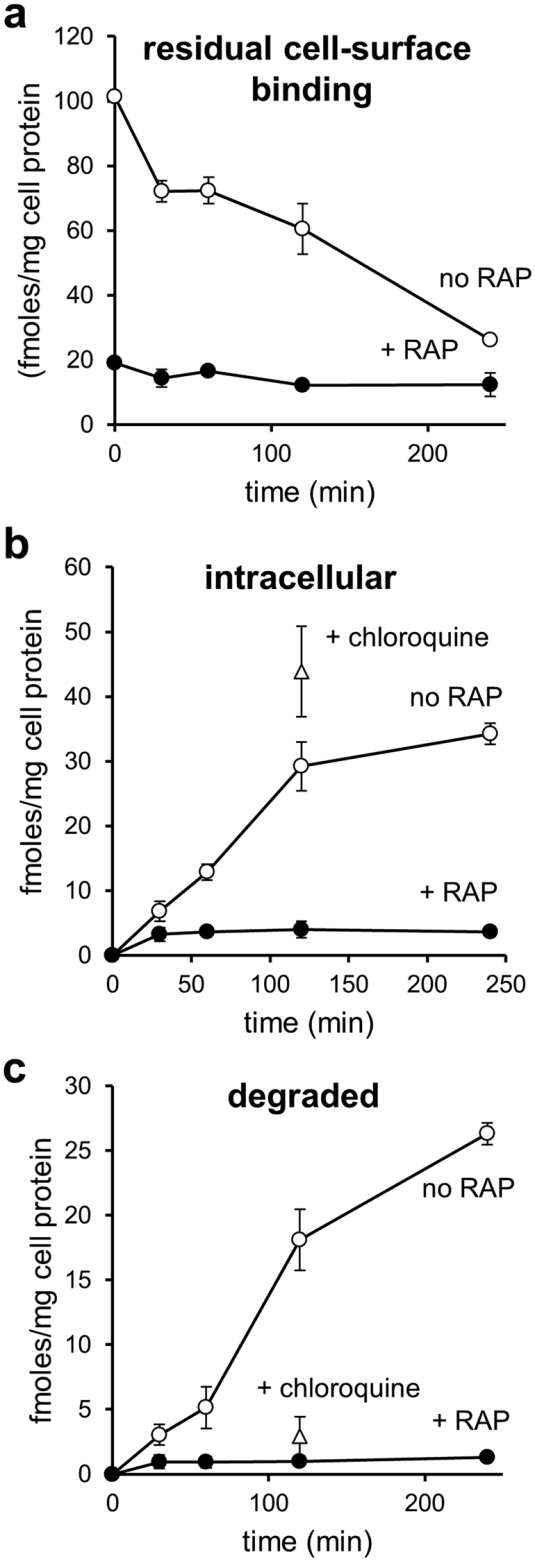
Uptake and degradation of ^125^I- proMMP-2:TIMP-2 complex by rat yolk sac BN16 cells. BN16 cells were incubated with 10 nM ^125^I-proMMP-2:TIMP-2 complex at 4 °C for 2 h to allow surface binding, in the absence (open circles) or presence of 1 μM RAP (closed circles). Some of the cultures were pre-treated with 100 μM chloroquine for 1 h at 37 °C (open triangles). After washing, cells were further incubated with fresh medium pre-warmed at 37 °C, without or with RAP or chloroquine as above. At the indicated times, the amounts of ligand (**a**) remaining surface-bound (pronase^®^-sensitive radioactivity), (**b**) internalized (pronase^®^-resistant radioactivity) and (**c**) degraded (trichloroacetic acid-soluble radioactivity in conditioned medium) were measured. Values are means ± S.D. of three dishes. This experiment was performed twice with similar results.

Once at 37 °C to allow endocytosis to proceed, cell-surface bound ^125^I-proMMP-2:TIMP-2 decreased progressively over time (Fig. 4a). Intracellular ^125^I-proMMP-2:TIMP-2 was maximal after 120 min (Fig. 4b). Intracellularly degraded ligand was progressively secreted back into the medium as trichloroacetic acid-soluble material and accumulated up to 240 min (Fig. 4c). Chloroquine treatment, which blocks lysosomal proteolysis by inhibiting endosomal acidification^19^, impaired degradation of ^125^I-proMMP-2:TIMP-2, (Fig. 4b,c), suggesting that intracellular breakdown of the complex was occurring in the lysosome. Altogether, these data indicate that megalin/LRP-2 can be an efficient receptor for endocytic clearance of the proMMP-2:TIMP-2 complex.

### Renal invalidation of megalin/LRP-2 expression in mice induces elimination of proMMP-2 and TIMP-2 in urine

**Figure 5:**
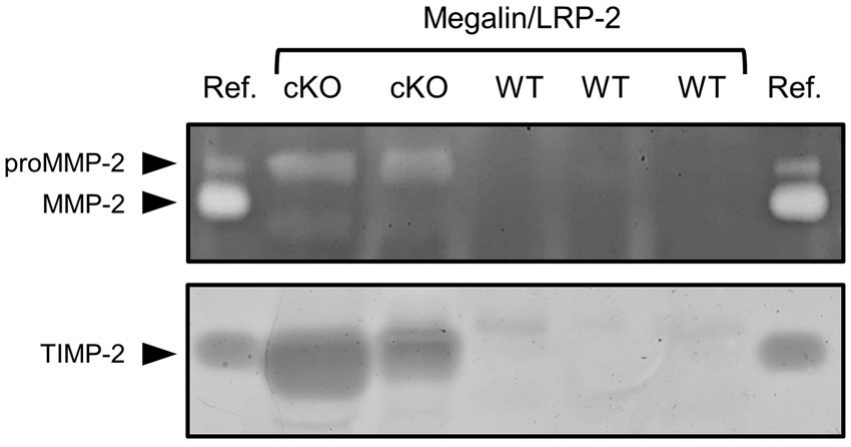
Invalidation of megalin/LRP-2 induces accumulation of proMMP-2 and TIMP-2 in urines. Twenty-four-hour urines were collected from 7-month-old *Meg* ^*lox/lox*^ *;Wnt4-Cre* ^*+*^ mice (cKO) or their wild-type littermates (WT) and samples were analyzed, without concentration, by direct (32 µl/lane; top panel) or reverse (16 µl/lane; bottom panel) zymography. Reference (ref.) corresponds to medium conditioned by mouse calvarium for direct zymography (as in Fig. 1) or 20 ng recombinant TIMP-2 for reverse zymography.

To determine whether megalin/LRP-2 can also mediate proMMP-2:TIMP-2 complex clearance *in vivo*, we used transgenic mice in which a conditional knockout of megalin expression (cKO) is induced by Wnt-4-driven Cre recombinase (*Meg* ^*lox/lox*^;*Wnt4-Cre* ^+^ mice)^17^. Megalin/LRP-2 is the major endocytic receptor for the tubular retrieval of filtered plasma proteins^20^ and megalin/LRP-2 cKO results in proteinuria. We therefore compared, by direct and reverse zymography, urines of cKO mice and of their wild-type littermates (Fig. 5). Megalin/LRP-2 cKO resulted in a strong accumulation of both proMMP-2 and TIMP-2 in the urines, suggesting that megalin/LRP-2 mediates renal reabsorption of these proteins.

## Discussion

In the present study, we show that megalin/LRP-2 acts as an endocytic receptor for proMMP-2:TIMP-2 complex. We found that RAP, an antagonist of the LDL receptor family^18^, competed with binding of proMMP-2:TIMP-2 complex onto rat BN16 epithelial cells. More specifically, anti-megalin/LRP-2 antibodies similarly inhibited binding of the complex. Moreover, megalin/LRP-2 was shown by surface plasmon resonance analysis to directly bind proMMP-2:TIMP-2, with a K_D_ in the 10^−8^ M range. Similar ranges were previously reported for the binding between megalin/LRP-2 and the proMMP-9:TIMP-1 complex^12^ as well as between LRP-1 and proMMP-2:TIMP-2^13^.

We further show that following cell-surface binding, proMMP-2:TIMP-2 complex is rapidly internalized by BN16 cells. We previously reported similar LRP-1-mediated endocytosis of proMMP-2:TIMP-2 in HT1080 cells. However, this involved initial RAP-insensitive binding of the complex to an unknown neighboring binding site at the plasma membrane^13^, whereas we report here a simple RAP-sensitive uptake mediated by megalin/LRP-2. Whether other similar complexes, such as proMMP-2:TIMP-4^21^, are also able to be internalized by LRP-1 or megalin/LRP-2-mediated endocytosis remains to be determined.

Although our data clearly show that megalin/LRP-2 directly binds proMMP-2:TIMP-2, it is possible that other membrane proteins acting as co-receptors, such as cubilin, contribute to internalization of the complex. Indeed, the megalin/cubilin receptor complex is important for physiological retrieval of various proteins and vitamins, as illustrated by studies in animal models such as the *Meg* ^*lox/lox*^;*Wnt4-Cre* ^+^ mice we used^17, 22, 23^. Deficient reabsorption of proMMP-2 and TIMP-2 by kidney-specific conditional KO mice underlines the major contribution of megalin/LRP-2. Since cubilin is still expressed in the kidney of the these cKO mice^17^, further studies are needed to clarify whether additional cubilin binding of proMMP-2 and TIMP-2 could occur *in vivo* prior to megalin-mediated internalization, as reported for albumin^24^.

Abrogation of complex degradation upon chloroquine addition^19^ strongly suggests that proMMP-2 and TIMP-2 are intracellularly degraded in a lysosomal-dependent process as previously evidenced for MMP-9^12^. However, additional studies would be interesting to analyze in more details the intracellular outcome of the ligands.

Our *in vivo* study highlights physiological relevance of megalin/LRP-2-mediated proMMP-2 and TIMP-2 uptake. In contrast, increased urinary concentration of proMMP-2 and/or TIMP-2 was measured in pathological situations involving kidney deficiency such as renal carcinoma^25–27^ and type 1 diabetic nephropathy^28^, suggesting reduced renal proMMP-2 and TIMP-2 reabsorption. Interestingly, it was recently reported that accumulation of MMP-7 in urine could play a role in the pathogenesis of kidney fibrosis^29^, suggesting that clearance of urinary MMPs may be important to preserve renal function. In addition, megalin/LRP-2 has been localized at the surface of type II pneumocytes of the lung alveoli^30^ where megalin/LRP-2-mediated endocytosis could thus represent a new mechanism for regulating levels of MMP-2 and MMP-9. Of note, these MMPs are largely increased in the airspace of patients with acute lung injury or acute respiratory distress syndrome^31^ as well as in idiopathic interstitial pneumonias^32^. Moreover, several MMPs and TIMPs, including MMP-2, MMP-9 and TIMP-2, are present in the semen^33, 34^. Furthermore, MMPs are found in the uterine fluid of buffalo^35^ and TIMP-4, an inhibitor of MMP-2 and MMP-9, is released into the uterine fluid^36^. Interestingly, megalin/LRP-2 is localized at the apical surface of the glands and surface epithelium in human endometrium^37^ and consequently is in contact of both semen and uterine fluids. Therefore, megalin/LRP-2 could protect the endometrium by regulating MMP activities from these fluids.

In conclusion, our study shows that megalin/LRP-2 directly binds the proMMP-2:TIMP-2 complex and is involved in its endocytic uptake, with potential physiological roles such as in the renal clearance.

## Materials and Methods

### Reagents

Dulbecco’s modified Eagle’s medium (DMEM), fetal calf serum (FCS), and other cell culture reagents were purchased from Invitrogen (Thermo Fischer Scientific, Erembodegem, Belgium). Na^125^I and Iodogen^®^ pre-coated tubes were from PerkinElmer Life Sciences and Pierce, respectively (both distributed by Thermo Fisher Scientific). Chloroquine was obtained from Sigma-Aldrich (Bornem, Belgium). Kaleidoscope SDS-PAGE molecular weight standards were from Bio-Rad Laboratories (Nazareth Eke, Belgium) and Himark^™^ prestained high-molecular-weight protein standard from Invitrogen. Pronase^®^, a mixture of proteinases isolated from the extracellular fluid of *Streptomyces griseus*, was from Roche Applied Science (Sigma-Aldrich).

### Proteins and antibodies

The proMMP-2:TIMP-2 complex was purchased from Enzo Life Sciences (Brussels, Belgium). Receptor-associated protein (RAP) was prepared as previously reported^38^. Polyclonal sheep antibodies anti-rat megalin/LRP-2 were a generous gift from Dr. R. Kozyraki (INSERM UMR S968, Paris, France). Monoclonal antibody anti-human LRP-1 85-kDa β-chain (mouse IgG2, clone 5A6) was from Oncogene (Calbiochem, San Diego, CA). Monoclonal antibody anti-glyceraldehyde phosphate dehydrogenase (GAPDH) (mouse IgG1, clone 6C5) was from Merck Millipore (Darmstadt, Germany). Blocking polyclonal rabbit antibodies anti-megalin/LRP-2 and anti-LRP-1 were from Biorbyt Ltd (Cambridge, United Kingdom) and Santa Cruz Biotechnology (Heidelberg, Germany), respectively. Non-immune rabbit IgGs, used as negative controls, were from Ancell (Bayport, MN). The specificity of anti-megalin/LRP-2 antibodies was assessed by western blotting using kidney homogenates from wild-type and megalin/LRP-2 cKO mice (Supplementary Fig. 1).

### Cell culture and RAP treatment

Brown Norway rat yolk sac carcinoma cells (BN16) were grown in DMEM supplemented with 10% (v/v) FCS, 100 IU/ml penicillin, 100 μg/ml streptomycin, 2 mM L-glutamine, and 25 mM HEPES buffer at 37 °C in a humid atmosphere (5% CO_2_ and 95% air). In experiments involving RAP treatment, subconfluent cells were cultured in the absence or presence of 1 μM recombinant RAP for 24 h in serum-free medium.

### Animal model

Megalin/LRP-2-deficient mice were generated using a conditional Cre-loxP system (*Meg* ^*lox/lox*^;*Wnt4-Cre* ^+^ mice), as previously described^17^. Mice were treated according to the National Institutes of Health Guide for Care and Use of Laboratory Animals. All procedures of the animal study were approved by the Ethical Committee for animal welfare at the Université catholique de Louvain (ref. 22). Twenty-four-hour urine samples were collected from seven-month-old mice in metabolic cages and analyzed, without concentration, by direct and reverse zymographies.

### Protein assay

Protein content was measured using the bicinchoninic acid microassay^39^, with bovine serum albumin as a standard.

### Western blotting

Western blotting was performed as previously described^13^ using 4–15% pre-cast gradient polyacrylamide gels. Cell extracts were normalized to protein content (50 μg/lane). Membranes were incubated overnight at 4 °C with primary antibodies diluted 1:1,000 (LRP-1 and -2) or 1:30,000 (GAPDH) in Tris-buffered saline (TBS: 50 mM Tris-HCl, 150 mM NaCl, pH 7.5) containing 0.1% Tween-20 and 5% dry milk powder. Immunoreactive bands were visualized using enzymatic chemiluminescence (Luminata Classico Western HRP substrate, Merck Millipore).

### Direct and reverse gelatin zymography

Samples of medium conditioned during twenty-four hours were normalized with respect to protein content, diluted in non-reducing 2% (w/v) SDS sample buffer and loaded onto 10% (for direct zymography) or 15% (for reverse zymography) polyacrylamide SDS gels containing 0.1% (w/v) gelatin. After electrophoresis, gels were washed at room temperature for 2 × 30 min in 2.5% (v/v) Triton X-100 to remove SDS and incubated at 37 °C for 16 h in reaction buffer (5 mM CaCl_2_, 1 µM ZnCl_2_, 0.2 mg/ml NaN_3_, 1% (v/v) Triton X-100 in 50 mM Tris-HCl buffer, pH 7.5). For reverse zymography, the reaction buffer was supplemented with 5% (v/v) medium conditioned by cultured mouse calvarium and treated with 0.4 mM 4-aminophenylmercuric acetate for 2 h at 25 °C to activate latent MMPs^37^, before incubation. After incubation, gels were stained for 30 min with 0.1% (w/v) G-250 Coomassie Blue in 45% (v/v) methanol, 10% (v/v) glacial acetic acid, and destained in the same solution without dye. Reverse zymography revealed inhibitory activity that appeared as dark zones against a clear background.

### Surface plasmon resonance analysis

The interaction of proMMP-2:TIMP-2 complex with megalin/LRP-2 was studied using a BIAcore 3000 instrument (BIAcore, Sweden). A CM5 sensor chip was activated with a 1:1 mixture of 0.2 M *N*-ethyl-*N*′-(3-dimethylaminopropyl)carbodiimide and 0.05 M *N*-hydroxysuccimide in water according to the manufacturer. Human megalin/LRP-2 was immobilized at 10 µg/ml in 10 mM sodium acetate, pH 4.0 at a density of 25 fmol/mm^2^. Remaining binding sites were blocked with 1 M ethanolamine, pH 8.5. A control flowcell was made by performing the activation and blocking procedure only. Ligands were diluted in the running buffer (100 mM Tris-HCl pH 7.4, 100 mM NaCl, 10 mM CaCl_2_ supplemented with 0.005% surfactant P20) and analyzed at concentrations from 10 to 250 nM. After each analysis cycle, sensor chips were regenerated using 1.6 M glycine-HCl buffer, pH 3.0. The BIAcore response, which is expressed in relative response units (RU), is the difference in response between megalin/LRP-2 and control flowcells. Kinetic parameters were determined by BIAevaluation 4.1 software using a Langmuir 1:1 binding model and simultaneous fitting of all sensorgrams.

### Radioiodination

ProMMP-2:TIMP-2 complex was labeled with ^125^I using Iodogen^®^ according to manufacturer’s recommendations. Specific activities ranged from 10 to 15 μCi/μg of complex. Trichloroacetic acid-precipitable radioactivity of used radioligands was always >97%.

### Radioligand binding assay

BN16 cells (5 × 10^5^) were plated onto 35-mm dishes and cultured to confluency, then washed twice with assay medium (DMEM containing 0.1% bovine serum albumin) and adapted to this medium at 4 °C for 1 h. Cells were then incubated or not with 1 μM RAP, 100 μg/ml polyclonal rabbit anti-megalin/LRP-2 or anti-LRP-1 antibodies or non-immune IgGs in assay medium at 4 °C for 30 min, prior to addition of 10 nM ^125^I-proMMP-2:TIMP-2 complex for 2 h. After careful rinsing (nine times with cold PBS, on ice), cells were surface-digested with 0.1% (w/v) pronase^®^ in DMEM at 4 °C to degrade surface-bound ligands and cause cell detachment. After cell collection by centrifugation, radioactivity released in supernatant (pronase^®^-sensitive), measured by γ−counting, was defined as surface-bound ligand. Binding of ^125^I-proMMP-2:TIMP-2 complex to plastic dishes without cells did not exceed 8% of total bound radioactivity.

### Endocytosis and degradation assays

Cells adapted to 4 °C were incubated with 10 nM ^125^I-proMMP-2:TIMP-2 complex in assay medium at 4 °C for 2 h, in the absence or presence of 1 μM RAP. After binding, cells were carefully rinsed nine times with cold PBS and further cultured in assay medium pre-warmed at 37 °C for the indicated times. To distinguish surface-binding from intracellular accumulation, cells were washed twice with cold PBS, and surface-digested with pronase^®^ as above. Radioactivity associated with pelleted cells (pronase^®^-resistant) was defined as internalized ligand. After precipitation by 10% (w/v) trichloroacetic acid and centrifugation of the conditioned medium collected before pronase^®^ treatment, radioactivity in the supernatant was taken to indicate the amount of degraded ^125^I-ligand. To inhibit lysosomal activity, 100 μM chloroquine was added to some cultures 2 h before radioligand binding. Internalization and degradation were then studied as above after 2 h at 37 °C, in the continued presence of chloroquine.

### Statistical analysis

Statistical significance was tested using Student’s t test for comparisons between cell culture conditions. Differences were considered as statistically significant at p < 0.05.

## Electronic supplementary material

Supplementary Figure 1
