# Supplementary material for: Cellular uptake of proMMP-2:TIMP-2 complexes by the endocytic receptor megalin/LRP-2
Source: Sci Rep. 2017 Jun 28;7:4328. doi: 10.1038/s41598-017-04648-y (PMC5489529; doi:10.1038/s41598-017-04648-y)
Supplement: Supplementary file 1 — Supplementary Figure 1 [file 41598_2017_4648_MOESM1_ESM.pdf]

Supplementary info

Cellular uptake of proMMP-2:TIMP-2 complexes by the endocytic receptor megalin/LRP-2

Manuel Johanns[1], Pascale Lemoine[1], Virginie Janssens[1], Giuseppina Grieco[1], Soren K. Moestrup[2], Rikke Nielsen[2], Erik I. Christensen[2], Pierre J. Courtoy[1], Hervé Emonard[3], Etienne Marbaix[1]* & Patrick Henriet[1]*§

[1]de Duve Institute, Université catholique de Louvain, 1200 Brussels, Belgium

[2]Department of Biomedicine, Aarhus University, 8000 Aarhus, Denmark

[3]CNRS UMR 7369, Matrice Extracellulaire et Dynamique Cellulaire, Université de Reims Champagne-Ardenne, 51687 Reims, France

*these authors contributed equally

§corresponding author: patrick.henriet@uclouvain.be

Supplementary Figure 1

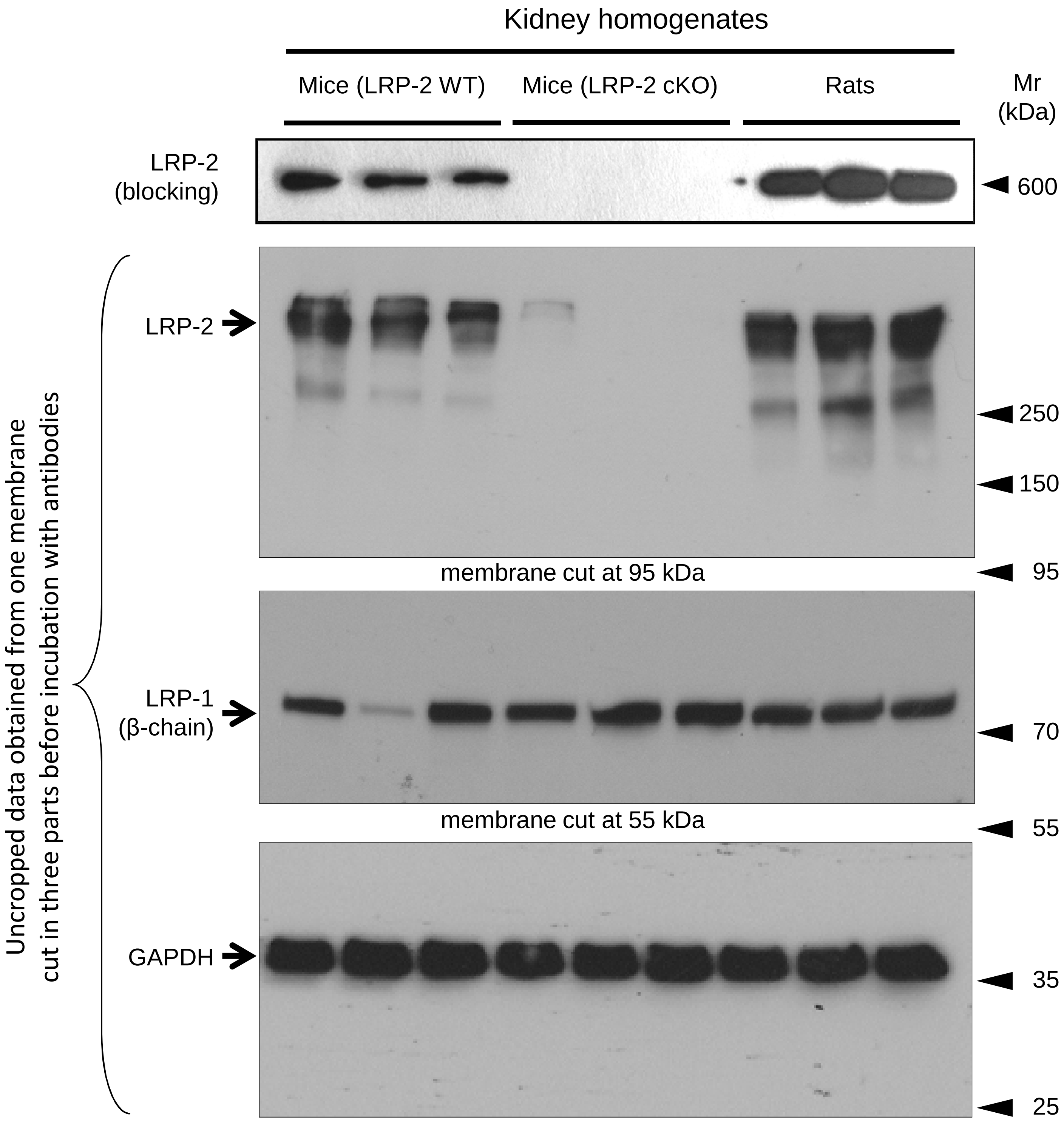

Supplementary Figure 1. Assessment of the specificity of the anti-megalin/LRP-2 antibodies. Kidney homogenates (50 µg protein) from megalin/LRP-2 cKO mice, their wild-type littermates, or wild-type rats were analyzed by western blotting using anti-megalin/LRP-2 antibodies used in Fig. 3 (blocking) or in Fig. 1, or the anti-LRP-1 antibody used in Fig.1. Please note that, unlike the LRP-1 signal, the megalin/LRP-2 signal almost disappears in samples from cKO mice by comparison with WT mice.
